# Situs inversus totalis with carcinoma of gastric cardia: a case report

**DOI:** 10.1186/1477-7819-10-263

**Published:** 2012-12-11

**Authors:** Pan Ke, Zhong Dewu, Miao Xiongying, Liu Guoqing, Jiang Qunguang, Liu Yi

**Affiliations:** 1 Department of General Surgery, Xiang-Ya 2nd Hospital, Central South University, Chang-Sha, Hunan Province, China

**Keywords:** Situs inversus, Carcinoma of the gastric cardia, Case report

## Abstract

Situs inversus is an uncommon anomaly with rare incidence. Some cases of situs inversus totalis have been described with different types of associations. Here we report a case of situs inversus with carcinoma of the gastric cardia.

## Background

Situs inversus is an uncommon anomaly with an incidence varying from 1 in 4,000 to 1 in 20,000 live births [1], with a male:female ratio of 3:2 [2]. Situs inversus viscerum can be either total or partial. Total situs inversus can be called a mirror image dextrocardia, characterized by a heart and stomach at the right side of the midline while the liver and gall bladder are at the left side. Although this does not seem to affect normal health or life expectancy, situs inversus viscerum only does not affect the patient’s health when it is premalignant. Generally, this rare genetic anomaly is discovered or diagnosed incidentally during thoracic and abdominal imaging. The exact etiology of situs inversus is still unknown, but an autosomal recessive inheritance mode has been speculated [3]. Some cases of situs inversus totalis have been described in previous studies [2,4-8]. We here report a case of situs inversus with carcinoma of the gastric cardia.

## Case presentation

A 52-year-old male patient, who complained of a stomachache lasting over the last 5 months, along with the symptoms of fatigue and weight loss, was referred to our clinic. An abnormal shadow in the cardia was recognized during routine X-ray examination and an upper gastrointestinal endoscopic examination. Upon admission, a physical examination revealed a pulse rate of 100 beats per minute and blood pressure of 115/68 mmHg, while the liver, spleen, and tumor were not palpable in the physical examination. Results of chest X-ray imaging taken in an erect position showed dextrocardia, a fundic gas shadow on the right dome of the diaphragm, and a liver shadow at the left side. Nothing abnormal was found in the respiratory system.

**Figure 1:**
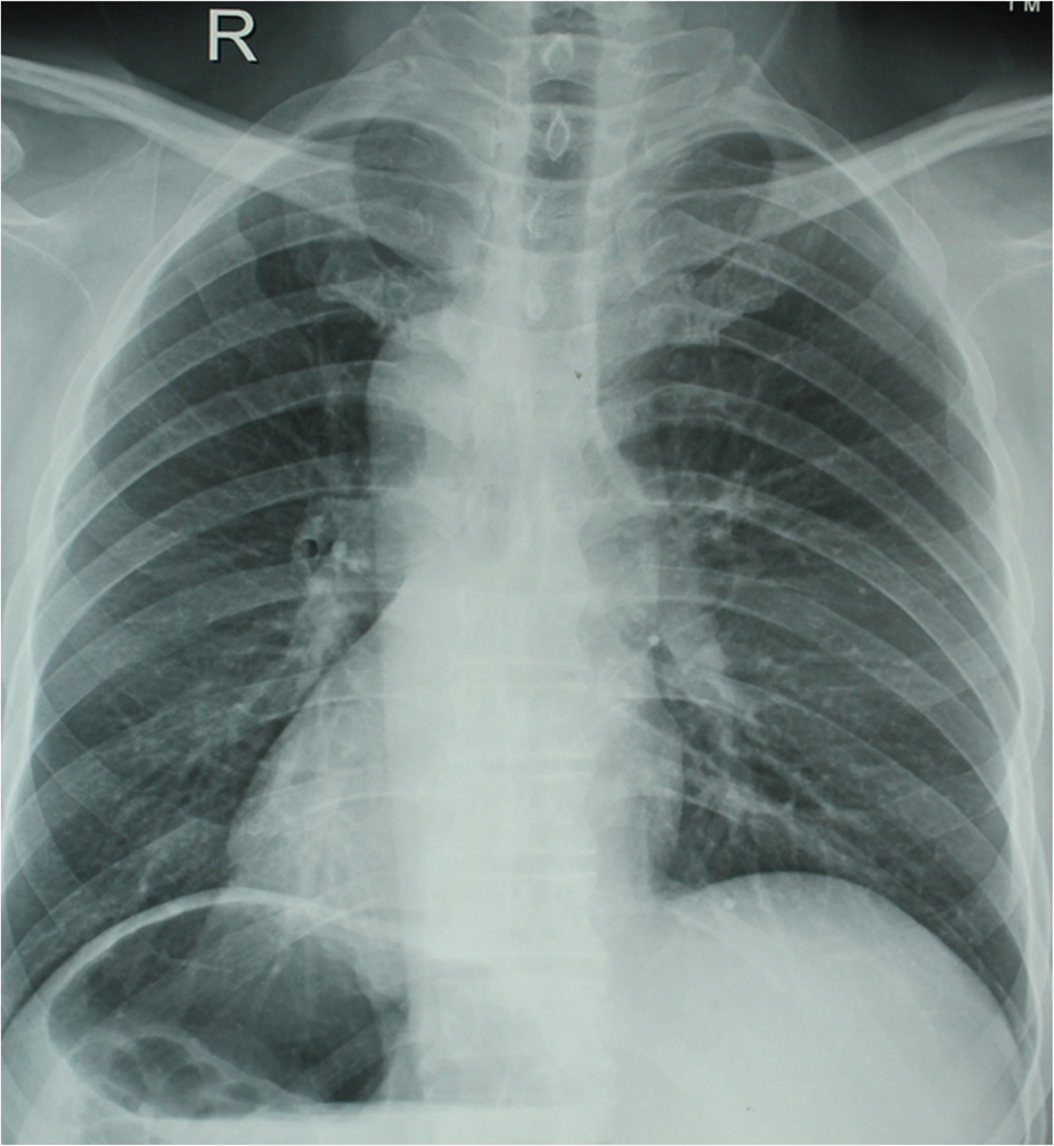
Chest X-ray demonstrating dextrocardia.

The laboratory findings were hemoglobin 11 g/dl and white blood cells 6,500/m^3^. Dextrocardia was seen on the chest X-ray (Figure 1), and a typical congenital dextrocardia was found on the electrocardiographic findings. The endoscopic examination identified a tumor in the gastric cardia. The pathological diagnosis was moderately differentiated and partly poorly differentiated adenocarcinoma. Surgery was performed on 25 July 2011, and macroscopic metastasis was found in lymph nodes 1, 2, 3, 7, 8a, and 8p (for the numbering rules, see [9]). The operative procedure was Billroth I reconstruction and proximal gastrectomy with D2 lymph node discectomy. The macroscopic findings of the resected specimen showed irregularly shaped gastric cardia of 20 mm × 18 mm size. The histological finding showed proliferation of moderately and poorly differentiated tubular adenocarcinoma, positive lymph vessels, and venous invasion. This was a type I adenocarcinoma of the esophagogastric junction (AEG) [10]. Metastases were recognized in lymph nodes 1, 3, and 8p in the sentinel lymph node biopsy. The patient’s postoperative course was uneventful; on day 15 after surgery, the patient was discharged from the hospital and commenced chemotherapy. 

## Discussion

Situs inversus is a positional anomaly that rotates the viscera of the internal abdomen, and it is a rare congenital anomaly with a population incidence of only 0.001% to 0.01% [11]. It is suggested that the immobility of nodal cilia inhibits the flow of extra-embryonic fluid during the embryonic period, and this leads to the development of situs inversus [12]. Its transmission mode is autosomal recessive inheritance, but its precise genetic mechanism is still unidentified. More than one genetic mutation including the gene mutations that cause ciliopathy and cystic renal diseases has been implicated in the etiopathogenesis. Various modalities can be used to diagnose situs inversus, such as electrocardiograms, radiographic studies, and computed tomography (CT) scans with oral and intravenous contrast, ultrasound, and barium studies [11,13].

There have been isolated reports of situs inversus associated with peptic conditions [14], ulcer perforation [15], amoebic liver abscess [8], acute cholecystitis [7,16], cholelithiasis [6,17,18], acute appendicitis [4,19], and intestinal obstruction [20]. The occurrence of carcinoma of the gastric cardia with situs inversus is very rare, and very few cases have been reported in the literature [5,21-24]. There are no data on the relationship between gastric cancer and situs inversus totalis. It is very important to carry out a careful and cautious assessment of abnormalities by preoperative examination, especially by laparoscopic procedures.

## Conclusion

The occurrence of gastric cardia with situs inversus is very rare. Proximal gastrectomy with lymph node dissection (D2) is the preferred operation for carcinoma of gastric cardia. Chemotherapy is essential for patients with lymph node metastasis.

## Consent

Written informed consent was obtained from our patient for publication of this case report and all accompanying images.

## Competing interest

The authors declare that they have no competing interests.

## Authors’ contributions

PK, ZD, MX, LG, JQ and LY designed the study, performed the data analysis and wrote the paper together; all authors read and approved the final manuscript.
